# Self-assembly of the chaperonin GroEL nanocage induced at submicellar detergent

**DOI:** 10.1038/srep05614

**Published:** 2014-07-08

**Authors:** Jin Chen, Hisashi Yagi, Yuji Furutani, Takashi Nakamura, Asumi Inaguma, Hao Guo, Yan Kong, Yuji Goto

**Affiliations:** 1 Okazaki Institute for Integrative Bioscience and Institute for Molecular Science, National Institutes of Natural Sciences, 5-1 Higashiyama, Myodaiji, Okazaki 444-8787, Japan; 2 Institute for Protein Research, Osaka University, 3-2 Yamadaoka, Suita, Osaka 565-0871, Japan; 3 Department of Life and Coordination-Complex Molecular Science, Institute for Molecular Science, Myodaiji, Okazaki 444-8585, Japan; 4 Department of Structural Molecular Science, The Graduate University for Advanced Studies (SOKENDAI), Myodaiji, Okazaki 444-8585, Japan; 5 State Key Laboratory of Materials-Oriented Chemical Engineering, Nanjing University of Technology, Nanjing 210009, China; 6 These authors contributed equally to this work.; 7 Current address: Department of Chemistry and Biotechnology, Graduate School of Engineering, Tottori University, 4-101 Koyama-minami, Tottori 680-8552 and Center for Research on Green Sustainable Chemistry, Tottori University, 4-101 Koyama-minami, Tottori 680-8552.

## Abstract

Protein nanoassemblies possess unique advantage in biomedical applications such as drug delivery, biocatalysis and vaccine development. Despite recent accomplishment in atomic structure data, the underlying molecular mechanism of protein self-assembly remains elusive, where considerable heterogeneity is often involved. Here we use *E. coli* chaperonin GroEL, a tetradecameric protein with a molecular weight of 805 kDa, to probe its transformation from cage-like oligomers to protein nanofibers. We show that sodium dodecyl sulfate (SDS), a widely-used protein denaturant, at submicellar concentration binds to and causes partial distortion of GroEL apical domain. Subsequently, the GroEL apical domain with altered secondary structural content converts the GroEL oligomers into modular structural units which are observed to self-assemble into cylindrical nanofibers under an agitated incubation in a physiological buffer. Interestingly, through targeted mutagenesis where two cysteine residues are introduced at the entry site of GroEL cage, we found that the formation of GroEL nanoassembly could be modulated depending on the redox condition of incubation. Without the need of chemical engineering, tunable GroEL nanofibers built by controlled-assembly are among the largest nanoscale bioassembly with broad applications.

Owing to its special advantages over other materials, protein nanofibers have attracted increasing attentions^1^,^2^,^3^,^4^,^5^,^6^. Besides the desirable biocompatibility, protein nanofibers often share a well-defined structural characteristics^7^, which demonstrate a large-scale modularity fulfilling the demand of biomedical applications. Moreover, such structural commonality has enabled numerous studies aimed at designing and synthesizing high-order protein-based structures^6^,^8^,^9^,^10^. Theoretical and experimental advance in determining structures of minimal built-up units or final nanoscale bioassembly at atomic resolution have provided evidence of structural determinants along their assembly-pathway^3^,^11^. However, the underlying molecular mechanism of nanoscale assembly of protein is poorly defined so far since transient intermediates as well as structural complexities are often involved with such process. We set out to use chaperonin GroEL, a naturally abundant tubular protein, to study its molecular mechanism of self-assembly.

Bacterial GroEL and its cofactor GroES are the most remarkable molecular chaperone system in *E. coli* whose function is to maintain the cellular homeostasis^12^,^13^. The GroEL oligomer consists of 14 identical subunits arranged into two stacked heptameric rings. Each GroEL protomer has three functional domains named as apical, intermediate and equatorial^14^,^15^. The apical domain harbors the binding site of substrate proteins and GroES. The intermediate domain is relatively flexible to flank equatorial and apical domain. The equatorial domain is responsible for most of intra-ring interactions and encloses a folding cavity for substrate proteins. To fulfill their function, GroEL and GroES form a nanocage-like structure^16^,^17^,^18^,^19^ to assist diverse substrate proteins to fold inside^20^,^21^,^22^.

## Results

### SDS binds to the GroEL apical domain

**Figure 1:**
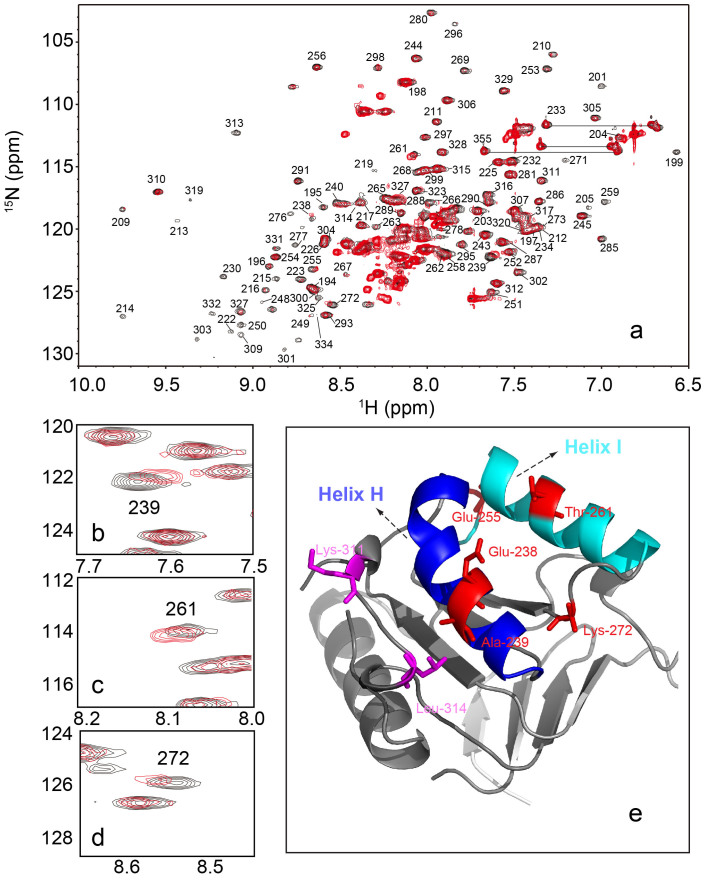
SDS binds to the substrate binding site of GroEL. (a) ^1^H-^15^N HSQC spectrum of 250 μM GroEL apical domain in the absence (black) or presence (red) of 0.1 mM SDS at pH 7.0 and 30°C. (b)–(d) Expanded regions of (a) showing residues in the GroEL apical domain with pronounced chemical shifts after the binding of SDS. The peaks were assigned as reported^24^. (e) Residues in b–d, Glu 238 and Glu 255 are labeled as red sticks in a portion of the GroEL apical domain. Two residues (K311and L314) for cysteine substitution are shown as magenta sticks.

We earlier found that the isolated GroEL apical domain (GroEL_191–376_, see Methods) can form amyloid-like fibrils in the presence of SDS^23^. Further study indicated that intact GroEL can also fibrillate under similar conditions^23^, suggesting a crucial role of SDS for the protein fibrillation. Note that the backbone assignments of a shorter apical domain construct (GroEL_191–335_) were previously resolved^24^. To study the binding mechanism of SDS to the GroEL apical domain, we performed the 2D-NMR analysis by using an isolated apical domain construct (GroEL_191–345_). Fig. 1 shows the resulting ^1^H-^15^N HSQC spectra of GroEL_191–345_ in the presence or absence of SDS. The overall 2D-NMR spectrum of GroEL_191–345_ showed well-resolved cross-peaks whose resonance positions are similar with that of GroEL_191–335_. Therefore, we are able to apply assignments of GroEL_191–335_ to GroEL_191–345_ to determine the SDS-binding sites. In general, most of cross peaks from two spectra are superimposible in the presence and the absence of SDS (Fig. 1), among which several cross peaks exhibit distinguishable chemical shift differences (^15^N, >±0.15 ppm or ^2^H, >±0.03 ppm^24^). We identified altogether five residues (Ala239, Thr261 and Lys272 were shown in Fig. 1b–d; Glu238, Glu255 were labeled as red sticks in Fig. 1e) which exhibit marked difference in the cross peaks of the 2D-NMR spectra as the SDS-binding sites. Interestingly, all these residues locate in the hydrophobic core of GroEL (Thr261 and Glu255 in helix I; Ala239 and Glu238 in helix H) (Fig. 1e) which was previously ascribed as substrate-binding sites^25^. This finding suggests that SDS binding-induced local structural changes in the region of GroEL apical domain might be crucial for the later assembly of proteins.

### Submicellar concentration of SDS induces the fiber formation of GroEL

**Figure 2:**
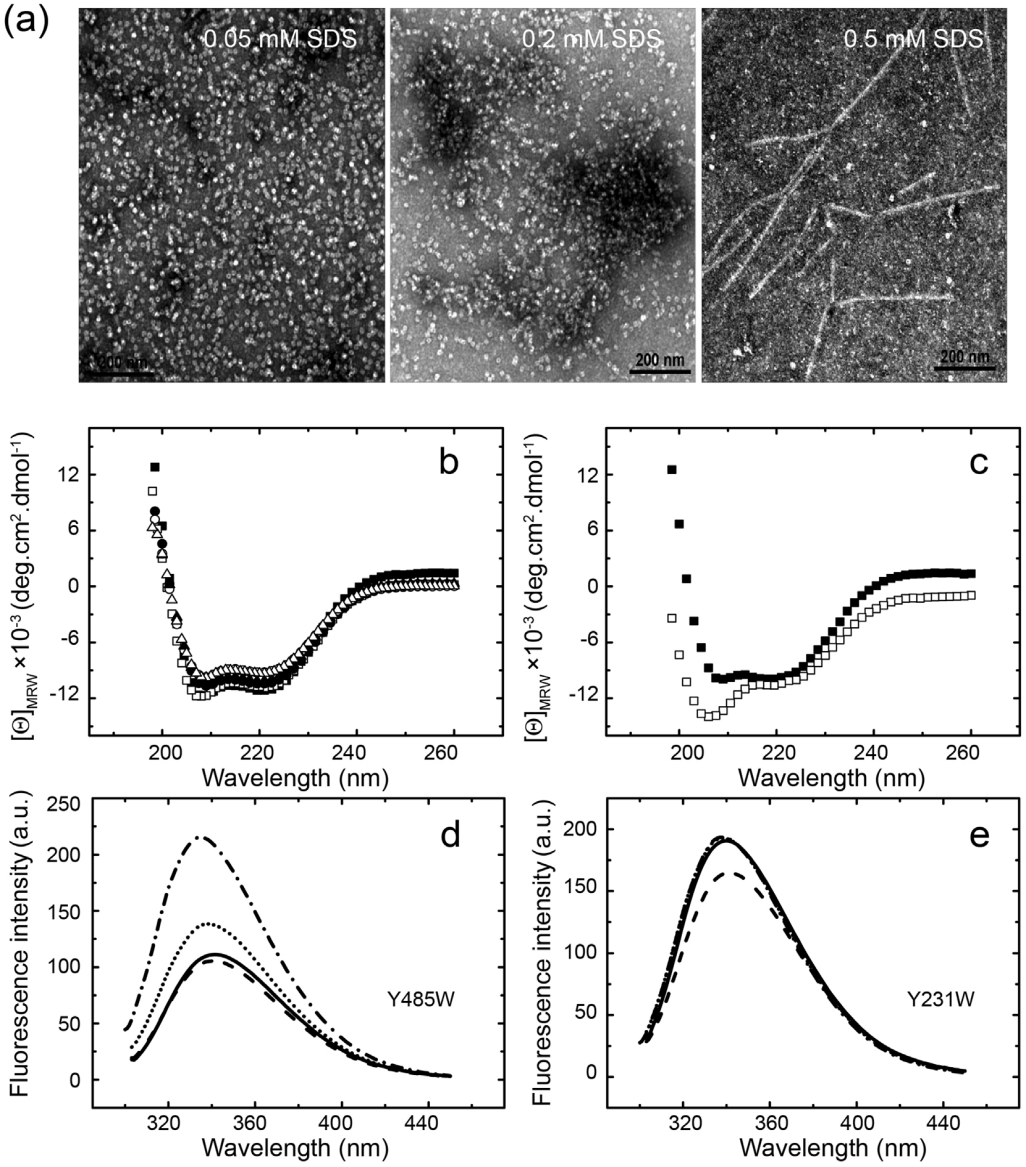
Submicellar concentration of SDS induces the nano-fiber formation of GroEL. (a), TEM observations of the nanofiber formation of GroEL at different concentration of SDS (incubated with agitation overnight). (b) Far-UV CD spectra of 50 μM wild-type GroEL, Y231W and Y485W in the presence (filled symbol) and absence (open symbol) of 1 mM SDS, respectively (wild-type GroEL: 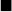 & 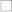; Y231W: 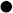 & 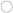; Y485W: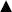 & 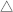). (c) Far-UV CD spectra of 40 μM GroEL apical domain in the absence (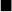) and presence (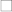) of 0.5 mM SDS. (d), (e) Tryptophan emission spectra of 5 μM Y231W and Y485W. Solid line: 0 mM SDS; dashed line: in 0.5 mM SDS; dotted line: in 1 mM SDS; dash-dot line: in 2 mM SDS.

The nanoscale assemblies of some typical aggregation-prone proteins often show a concentration-dependence of SDS^26^,^27^ due to the micelle formation of surfactants including SDS above certain concentration (defined as CMC, *critical micelle concentration*)^28^. We thus used transmission electron microscopy (TEM) to study the effect of SDS concentration on the formation of GroEL nanofibers. As visualized in TEM observation (Fig. 2a), the formation of GroEL nanofibers shows an evident concentration-dependence of SDS. At 0.05 and 0.2 mM SDS^29^, there is virtually no fiber formation of GroEL (Fig. 2a). When SDS concentration is increased to 0.5 mM close to its critical CMC^27^, an apparent fiber formation of GroEL is observed (Fig. 2a), indicating that SDS at submicellar concentration is crucial for the nanoscale assembly of GroEL.

We further study the accompanying secondary structural changes of protein samples caused by SDS-binding by far-UV CD spectroscopy. The overall CD spectra of wild-type GroEL as well as Trp mutants (R231W and Y485W) are overlaid to each other in the absence and presence of SDS (Fig. 2b). By contrast, 0.5 mM SDS introduces a pronounced secondary structural change to the GroEL_191–345_ (Fig. 2c) which is evident from the increase of the negative peak around at 207 nm. The observation means that the fraction of α-helix structure increases in the presence of 0.5 mM SDS, which is consistent with our previous result^23^ and surface-enhanced infrared absorption (SEIRA) observation as shown later. These findings suggest that SDS-binding to GroEL induces a local structural change for the intact protein^27^,^30^. This result is further corroborated by recording fluorescence spectra of two GroEL Trp mutants, R231W and Y485W, which were originally introduced to study structural change in GroEL upon ATP-binding^31^,^32^. Therefore, we hypothesize that by using these two mutants we are able to monitor the SDS binding-induced local structural changes of GroEL around ATP-binding site and apical domain, respectively. As shown in Fig. 2d, the fluorescence signal of Y485W, a Trp substitution in the equatorial domain around the ATP binding site (supplementary Fig. 1), shows a SDS concentration-dependent increase of fluorescence intensity. As Trp485 locates in the exterior of the GroEL oligomer, we reason that such fluorescence signal increase may attribute to the solvent effect or the localized structural changes^33^. By contrast, 0.5 mM SDS brings relatively marked fluorescent signal changes to R231W (Fig. 2e), where a hydrophilic residue of Arg in the GroEL apical domain was replaced (supplementary Fig. 1). At higher concentration of SDS (>0.5 mM), the fluorescent spectrum of R231W is virtually identical to that in the absence of SDS (Fig. 2e). As 0.5 mM SDS is close to its critical CMC value in solution, we conclude that 0.5 mM SDS may cause more pronounced structural changes in the region of apical domain of GroEL ready for the assembly of protein.

### Monitoring SDS-incuced structural changes of GroEL apical domain by SEIRA

**Figure 3:**
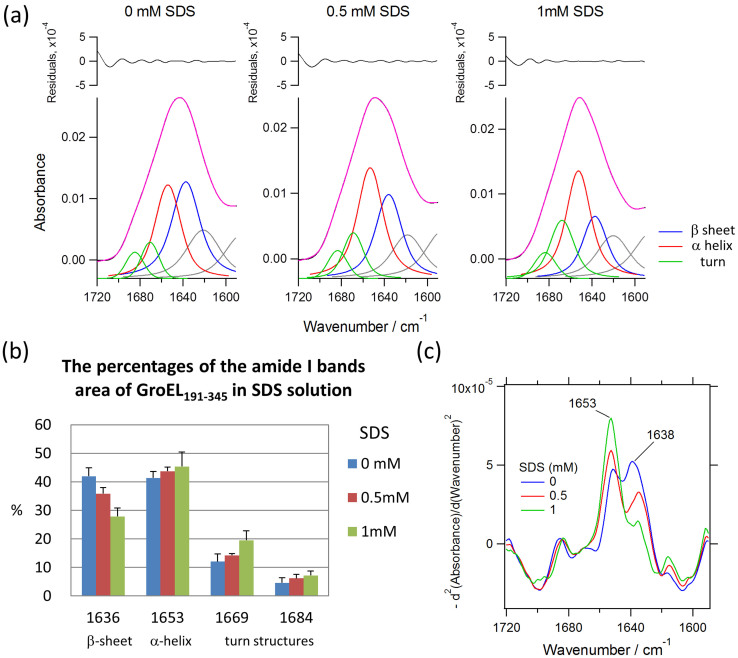
SDS-induced structural changes of the GroEL apical domain probed by SEIRA spectroscopy. (a) SEIRA spectra of 50 mM GroEL_191–345_ in the absence (0 mM SDS), and presence of 0.5 mM and 1.0 mM SDS. The experimental data (black line) were fitted with 6 Voigt function components as indicated (magenta). The residuals are shown in the top of the graphs. The bands at 1636 (blue), 1653 (red), and 1669 and 1684 (green) are corresponds to the α-helix, β-sheet and turn structures, respectively. (b) The second derivatives of the experimental data calculated after smoothing treatments. The SDS concentration of each spectrum is as indicated. (c) The percentages of the amide I bands area of GroEL_191–345_ in SDS solution. Each data was obtained from three independent experiments and the standard deviations were shown in the graph. The amide I bands at 1636, 1653, 1669 and 1684 cm^−1^ were resolved by the curve fitting as shown in (a).

The molecular elucidation of the protein nanoassembly is often hampered by its heterogeneous processing which may be difficult to be accessed by conventional methods. To circumvent this problem, we employ surface-enhanced infrared absorption (SEIRA) spectroscopy to probe protein conformational changes required for assembling by using the shorter GroEL_191–345_ construct. This technique allows for the detection of structural information from sub-monolayer protein films, which results from the enhanced electromagnetic field and therefore IR signal in the vicinity of gold nanostructures^34^. The tactic of gaining the SEIRA spectroscopy of GroEL_191–345_ is depicted in supplementary Fig. 2. Briefly, a gold film with 7 nm thicknesses was deposited onto a silicon prism, which served as an optical element in attenuated total reflection (ATR) configuration. The microscopic morphology of gold film is also visualized by the atomic force microscopy (AFM), which demonstrates a worm-like gold islands structure with irregular voids among them^35^. From the previous report, the thickness (7 nm) and morphology (worm-like structure) of the gold film is important for sufficient enhancement of infrared absorption of adsorbed proteins with less distorted spectral shape^35^. After the adsorption of GroEL_191–345_ on the gold film reached a steady state (~90 min), SEIRA spectrum was recorded in 50 mM phosphate buffer (pH 7.0) as a reference spectrum. The immobilization was evidenced by two prominent bands at 1641 and 1545 cm^−1^, which are assigned to the amide I and amide II vibrational modes of the protein backbone, respectively. The secondary structure content of adsorbed GroEL_191–345_ was identified by the amide I band (1700–1600 cm^−1^) analysis. As shown in Fig. 3a, Curve-fitting approaches were used to separate individual subcomponent bands overlapped in the amide I envelope (the black curve represents the original spectrum and the magenta one overall fit with 6 Voigt functions shown in the bottom of the graph). Component bands near 1636 cm^−1^ can be assigned to β-sheet; the band near 1669 and 1680 cm^−1^ to β-turn; the band near 1653 cm^−1^ to α-helix; and the band near 1620 cm^−1^ to side chain vibrations^36^. Assuming the areas of the former four component bands offer total IR absorbance in the amide I region, percent area, i.e., secondary structure content, of each band can be calculated (Fig. 3b). Three independent results revealed that the structure of adsorbed GroEL_191–345_ is 42 ± 3% β-sheet, 41 ± 2% α-helical, and 17 ± 3% β-turn structures. As a comparison, the SEIRA data from the adsorption of GroEL_191–345_ with 0.5 mM and 1 mM SDS (Fig. 3a) was obtained and handled following the procedure described above. Analysis showed that the contents of β-sheets, α-helices, and β-turns in 0.5 mM SDS are 36 ± 2, 44 ± 1 and 20 ± 2%, respectively. The contents in 1 mM were estimated to be 28 ± 3, 45 ± 5, and 27 ± 4%, respectively. Fig. 3b compares the fraction of the secondary structure of surface tethered GroEL_191–345_ in the presence of 0, 0.5, and 1 mM SDS. It can be seen that the addition of SDS systematically decreases the β-sheet structure and increases the α-helix and β-turn structures. Furthermore, such feature was also confirmed by the second derivative spectra of the original data (Fig. 3c) and the difference spectra calculated before and after addition of SDS (supplementary Fig. 3). From the analysis of the difference spectra, we estimated the time constants for reaching the equilibrium states in 0.5 and 1 mM SDS to be 19 and 9.6 min, respectively. Therefore, based on the quantitative analysis of SEIRA data, we conclude that the submicellar SDS leads to a β-sheet deformation of the apical domain of GroEL, which account for a self-assembly mechanism of protein nanocage.

### Tunable GroEL nanofiber

**Figure 4:**
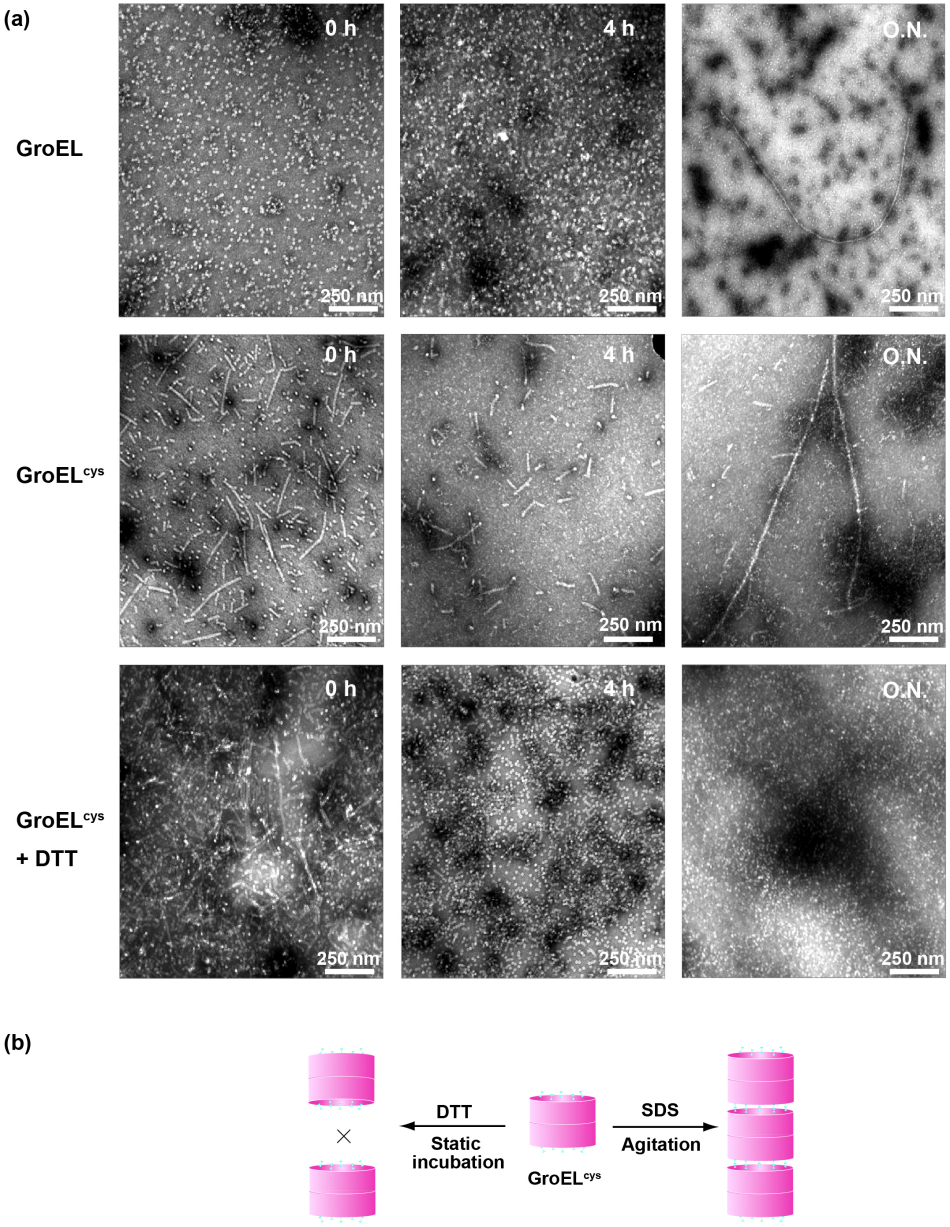
Tunable GroEL nanofiber. (a). TEM observations of the nanofiber formation of GroEL and GroEL^cys^ in the absence and presence of DTT. (b). A schematic figure of the self-assembly of GroEL nanofiber.

Recent report indicate that GroEL nanofibers prepared by chemical engineering exhibit remarkable mechanical stability^37^, which suggests the modularity of the intact protein. We ask whether our produced GroEL nanofibers could be modulated using target mutagenesis. To this end, we introduce two cysteine mutation at the entry sites of the protein cavity (GroEL^cys^: C → A; K^311^ → C, L^314^ → C)^37^ and study its fibrillation. As visualized by TEM measurement, the wild-type GroEL samples form apparent cylindral nanofibers after overnight incubation (Fig. 4a). By contrast, GroEL^cys^ samples contain significant amount of relatively shorter nanofibers even at the beginning of incubation and relatively longer fibers start to form after overnight incubation (Fig. 4a). Interestingly, when agitation is removed, cylindral fiber formation of GroEL is greatly diminished over the incubation period, which is getting more evident when DTT is added to the incubation solution (Fig. 4a and supplementary Fig. 4) suggesting a determining role of agitation to induce the fiber formation of protein. These results indicate that GroEL assembling process could be regulated by the site-specific mutation in its apical domain, which may act as the interfacial region of higher supramolecular assemblies^38^. The essential role of apical domain for GroEL self-assembly is further supported by the experimental observation that wild-type GroEL instead of single-ring GroEL could form nanofibers under the same condition (supplementary Fig. 5). Taken together, a molecular mechanism of GroEL nanofiber formation could be postulated. Depending on the redox condition of incubation, the assembling of GroEL nanocage can be tunable through target mutation at the interface of protein cavity (Fig. 4b), where the oxidized incubation (agitation) apparently accelerate the GroEL nanofiber formation but reducing condition (DTT added and no agitation) halted it to a great extent.

## Discussion

To elucidate the molecular mechanism by which bacterial chaperonin GroEL assembles into protein nanotubes, we employ multidisciplinary approaches including 2D-NMR, equilibrium measurement, electron microscopy and SEIRA to characterize its associated conformational changes. The ensemble experimental results revealed a SDS binding-induced structural change in GroEL substrate binding sites, i.e. apical domain, which may promotes the later nanoscale assembly of GroEL.

The GroEL apical domain was found to be the aggregation-prone region^23^ which the intact protein adopt to fulfill diversely cellular function. Such fibrillogenic propensity for the GroEL may be greatly depressed due to the multiple molecular interactions imposed in the cell. Nevertheless, submicellar concentration of SDS may cause a partial exposition of hydrophobic core and promotes the β-sheet deformation of GroEL apical domain as revealed by SEIRA measurement. This local structural conversion appears necessary for the self-assembly of GroEL because in contrast to the single-ring mutant, wild-type GroEL forms apparent cylindral fibers in the presence of SDS (supplementary Fig. 5), suggesting the importance of apical domain as a structure motif. This mechanism is further supported by the target mutagenesis on the apical domain which results in a controlled protein-assembly.

Previous methods to produce protein nanofibers rely much on labor-intensive or costly procedures^10^,^37^. In this study, we succeed in using bacterial GroEL, a naturally abundant barrel-like protein, to produce protein nanotubes under a much milder condition. For a long-term application purposes, our method offers an alternative for the controlled self-assembly of proteins to produce novel biomaterials.

As GroEL could fibrillate under physiological conditions, a question arises as what are the accompanying physiological function. Despite the unidentified functionality of supramolecular assembly, several implications can be postulated. Firstly, as SDS is reminiscent of cellular membranes in some of their characteristics, the phenomenon of GroEL fibrillation may account for a role of its gain-of-function, where co-aggregation with substrate proteins often occurs to respond the environmental stimulus^39^. Recently, the chapernoin originating from hot springs exhibits apparent fibrillar structures which resemble cytostructures^40^. Therefore, our study may provide detailed structural evidence correlating with some biological functions for chaperonins under stressed conditions.

## Methods

### Proteins preparation and nanofiber formation

GroEL tryptophan-substituted mutants (Trp-GroEL: R231W, Y485W) were constructed using site-directed mutagenesis. Cloning was performed using the Takara PrimeSTAR mutagenesis basal kit (TaKaRa). The isolated GroEL apical domain (GroEL residue 191–345^23^,^24^,^41^, denoted as GroEL_191–345_ in the text) was cloned into a PET24a vector (Invitrogen, Carlsbad, CA) yielding an expression plasmid with an N-terminal histidine-tag. At our hands, GroEL_191–345_ showed better expression than GroEL_191–335_, so we used GroEL_191–345_ in this study. All constructs including wild-type GroEL, Trp-GroEL and cysteine substituted GroEL mutant (GroEL^cys^: C → A; K311 → C, L314 → C) bearing 14 Cys residues^37^ at the entry site of protein cavity were expressed in *E. coli* BL21 (DE3) cells, and the proteins were purified to homogeneity as described previously^23^,^42^,^43^. The His-tagged GroEL apical domain was expressed in *E. coli* BL21 (DE3) cells and was uniformly enriched with ^15^N in M9 minimal medium containing ^15^NH_4_Cl (Sigma) as reported^24^. Protein quality was verified by SDS polyacrylamide gel electrophoresis with both coomassie-brilliant blue and silver staining^32^.

To investigate the nanofiber formation of protein samples, we employed optimized incubation conditions as reported^23^ in this laboratory. Briefly, 0.6 mg/ml GroEL samples as well as its mutants (GroEL^cys^) were incubated in a physiological buffer (sodium phosphate) at pH 7.0 in the presence of SDS. As agitation of stirring or shaking may accelerate the fibrillation process a protein, the fiber formation of protein samples were examined under agitated incubation in this study if not specified elsewhere.

### Fluorescence and Far-UV circular dichroism (CD) spectroscopy

SDS-binding induced conformational changes of GroEL was measured using a Jasco FP-6500 spectrofluorometer. The assay temperature was kept constant at 20°C using a water bath. The final concentration of protein was 0.5 μM. Far-UV CD spectra were measured in a 0.2-mm quartz cuvette on a Jasco J-720 spectropolarimeter equipped with a constant-temperature water bath. The standard buffer used in the measurements contained 50 mM sodium phosphate and 0.1 M NaCl in the absence or presence of SDS at pH 7.0.

### ^1^H-^15^N NMR spectroscopy

All NMR spectra were recorded on a Bruker AVANCE 500 MHz NMR spectrometer. We measured ^1^H-^15^N HSQC spectra of 250 μM GroEL apical domain in the presence or absence of 100 μM SDS at pH 7.0 and 30°C. We acquired 16 transients for each of 112 *t*1 points, and the sweep widths in *t*1 and *t*2 were 1723 and 6010 Hz, respectively. All NMR spectra were processed and analyzed by NMRPipe and NMRView.

### Electron microscopy

The microscopic structures of GroEL nanofibers formed at specified conditions were observed using a HITACHI H-7650 transmission microscope (Hitachi, Tokyo, Japan) operated at 80 kV. 5 μl samples were placed on a 400-mesh copper grid covered by a carbon-coated colloidal film for 60 s. Grids were negatively stained with 5 μl of 2% (w/v) uranyl acetate solution for 60 s. Excess sample solutions were removed with filter paper. The magnification was set to 10,000–30,000.

### SEIRA

The experimental procedures for the preparation of gold films and surface modification have been described elsewhere^35^. Briefly, a 7-nm-thick gold film was prepared on the silicon ATR prism by vacuum deposition at a deposition rate of 0.005 nm/s. A Ni-NTA monolayer was formed on the gold surface, according to the previous report^44^, by successively immersing the gold film in solutions of dithiobis(succinimidylpropionate) (Thermo Scientific) in dimethyl-sulfoxide (Nacalai Tesque, Inc.), amino-nitrilotriacetic acid (Dojindo Laboratories Inc.) in 0.5 M KHCO_3_ buffer, and NiSO_4_ in acetate buffer. Attachment of His-tagged GroEL_191–345_ was achieved by exposing the modified gold film to a 50 μM GroEL_191–345_ in 50 mM phosphate buffer solution (pH 7.0). The spectrometer was a Bruker Vertex 70 with a mercury cadmium telluride (MCT) detector. For each SEIRA spectrum, 128 scans were averaged with a 4 cm^−1^ resolution by a VeeMAX II ATR accessory. The incident angle was 48°. An IR polarizer was introduced between the detector and ATR and p-polarized light was used. The obtained spectra were analyzed by Igor Pro (ver 6.34) software. The spectra were offset at 1720 cm^−1^ and normalized at 1544 cm^−1^, where the peak of the amide II band is located. Multi-Peak Fitting (ver 1.4) was applied for decomposing the spectra in the amide I region into 6 Voigt functions, which is the convolution between a Gaussian and a Lorentzian function. The second derivatives were calculated after smoothing the original spectra by using algorism of Savitzky-Golay with 2^nd^ order.

## Author Contributions

J.C., H.Y. and Y.G. designed the research. J.C. and K.Y. prepared samples and performed the fluorescence and CD measurement. H.Y. performed the fiber measurement. A.I., H.G. and Y.F. performed the SEIRA analysis. J.C. and T.N. performed the NMR measurement. J.C., H.Y., H.G., Y.F. and T.N. wrote the manuscript. All authors discussed and improved the manuscript.

## Supplementary Material

Supplementary Information Dataset 1
