# Supplementary material for: Self-assembly of the chaperonin GroEL nanocage induced at submicellar detergent
Source: Sci Rep. 2014 Jul 8;4:5614. doi: 10.1038/srep05614 (PMC4085630; doi:10.1038/srep05614)

Supporting information

Self-assembly of the Chaperonin GroEL nanocage induced at submicellar detergent

Jin Chen[1*], Hisashi Yagi[2,*#], Yuji Furutani[3,4], Takashi Nakamura[1], Asumi Inaguma[3], Hao Guo[3,4], Yan Kong[5], Yuji Goto[2]

[1]Okazaki Institute for Integrative Bioscience and Institute for Molecular Science, National Institutes of Natural Sciences, 5-1 Higashiyama, Myodaiji, Okazaki 444-8787, Japan
[2]Institute for Protein Research, Osaka University, 3-2 Yamadaoka, Suita, Osaka 565-0871, Japan
[3]Department of Life and Coordination-Complex Molecular Science, Institute for Molecular Science, Myodaiji, Okazaki 444-8585, Japan
[4]Department of Structural Molecular Science, The Graduate University for Advanced Studies (SOKENDAI), Myodaiji, Okazaki 444-8585, Japan
[5]State Key Laboratory of Materials-Oriented Chemical Engineering, Nanjing University of Technology, Nanjing 210009, China

*These authors contributed equally to this work.

# Current address (H.Y.): Department of Chemistry and Biotechnology, Graduate School of Engineering, Tottori University, 4-101 Koyama-minami, Tottori 680-8552 and Center for Research on Green Sustainable Chemistry, Tottori University, 4-101 Koyama-minami, Tottori 680-8552

Correspondence and request for materials should be addressed to J.C. (email: okachen30@gmail.com)

Supporting information

Figure S1 | Two trp mutants used for tryptophan fluorescence spectroscopy measurment. (**a**) A GroEL protomer showing 2 tryptophan mutants in GroEL structural domains (apical is magenta, intermediate is green and equatorial is blue; mutated residue shown as red stick). (**b**)-(**c**) Expanded region circle-shaded in **b** showing a portion of GroEL structure around substrate binding site (b) and nucleotide-binding site (c).

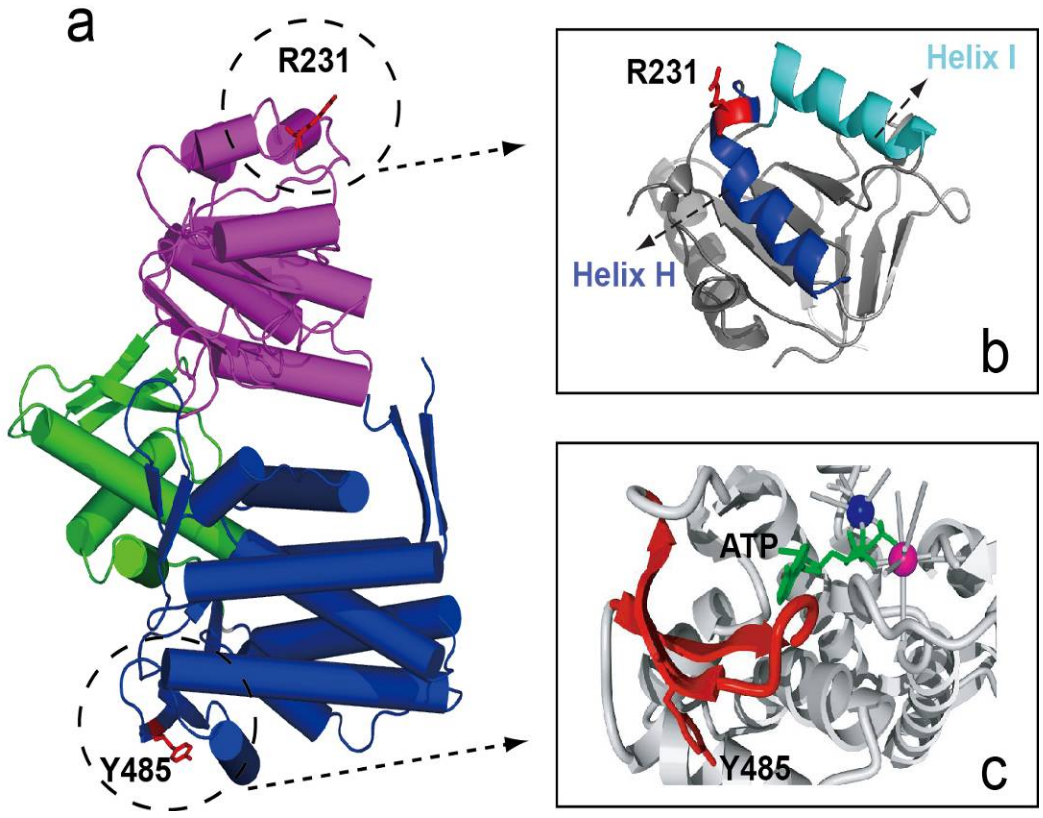

Figure S2 | A schematic illustration of the interface construction for SEIRAS detection of His-tagged protein.

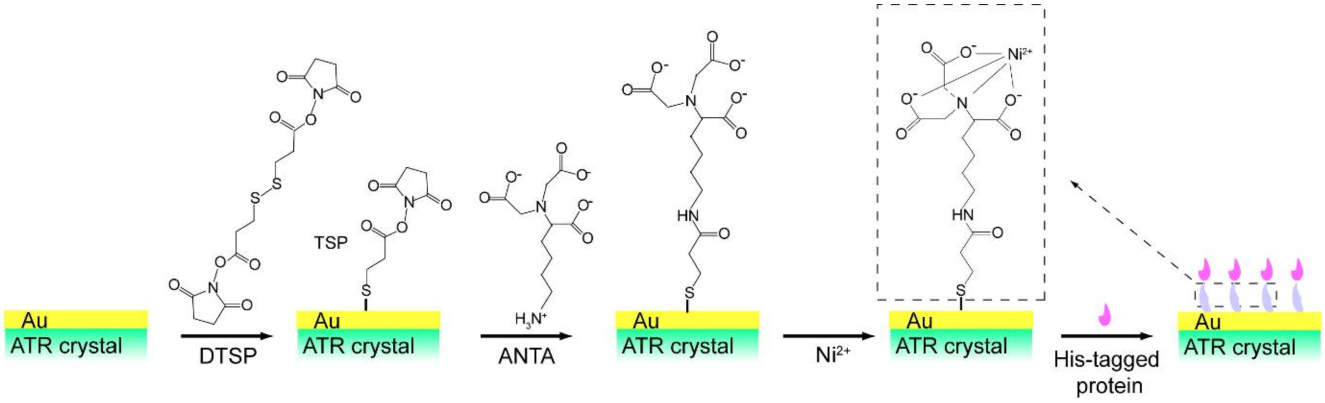

Figure S3 The kinetic data for SEIRA experiments on the effect of SDS on GroEL apical domain (**a**) SEIRA spectra of $GroEL_{191-345}$ recorded after replacing a buffer solution with 0.5 mM SDS or 1 mM SDS solution. (**b**) The difference spectra calculated by subtracting the SEIRA spectra measured in the absence of SDS from those after addition of SDS solution in the specified concentration (red; 0.5 mM, blue; 1 mM). (**c**) The plots of the difference absorbance at 1638 (red circle) or 1632 cm^{-1} (blue rectangle). The data for 0.5 mM and 1 mM SDS conditions were fitted by a single exponential with a time constant of 19 and 9.6 min, respectively.

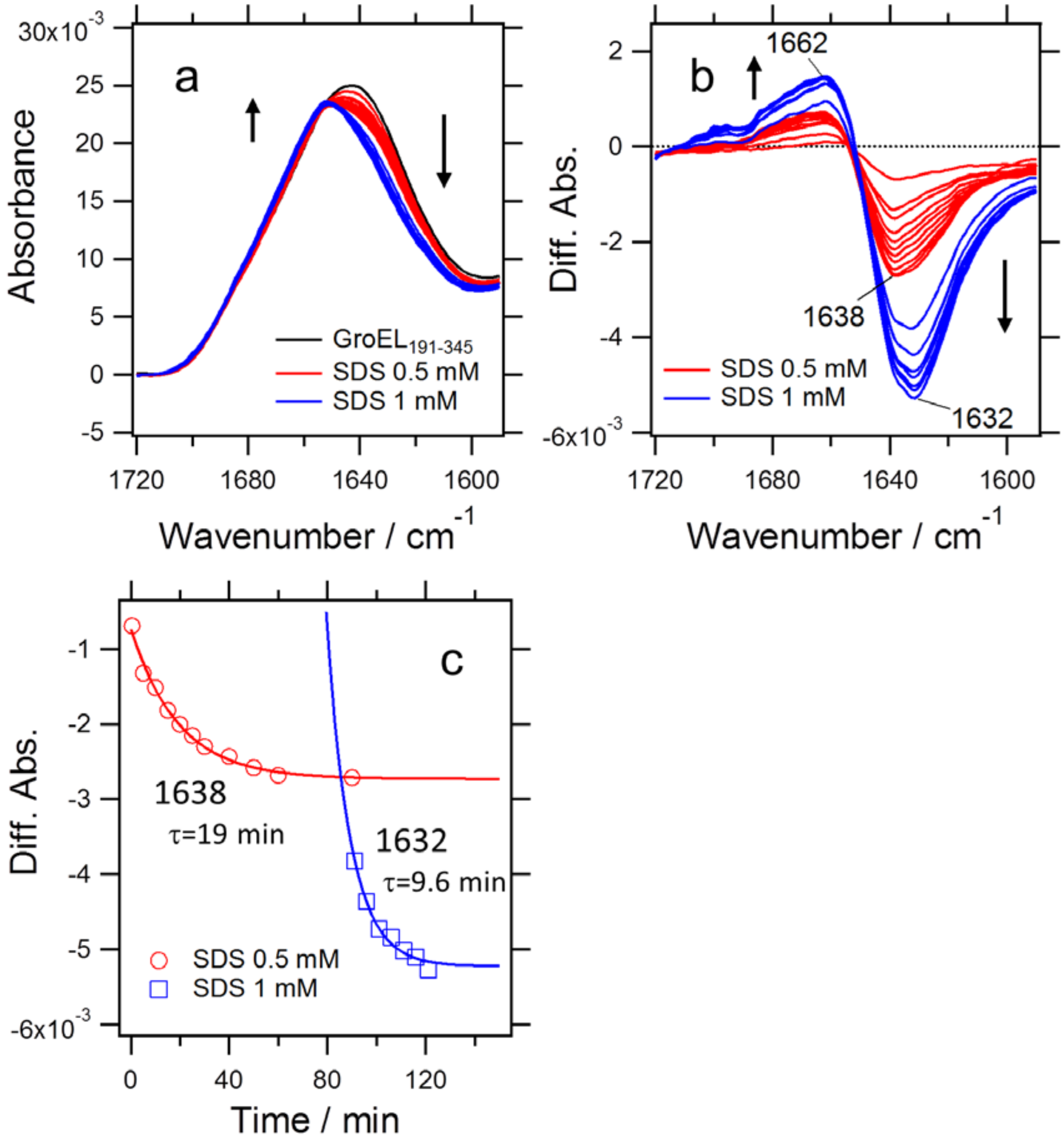

Figure S4 | TEM observation of the nano-fiber formation of GroEL^{cys} under different experimental conditions (incubated over night). Two representative pictures were presented.

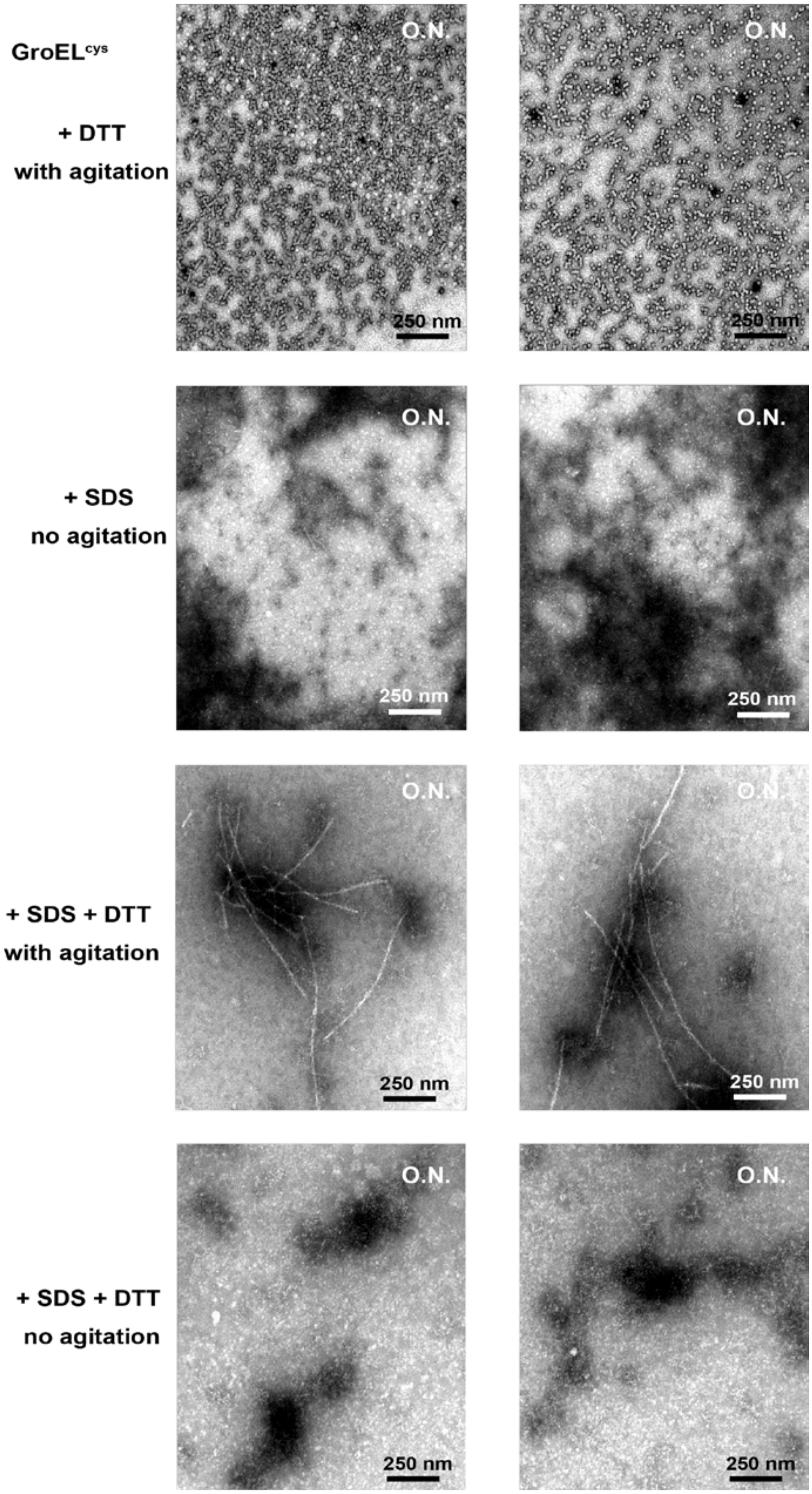

Figure S5 | TEM observation of the nano-fiber formation of wide-type and single-ring GroEL.

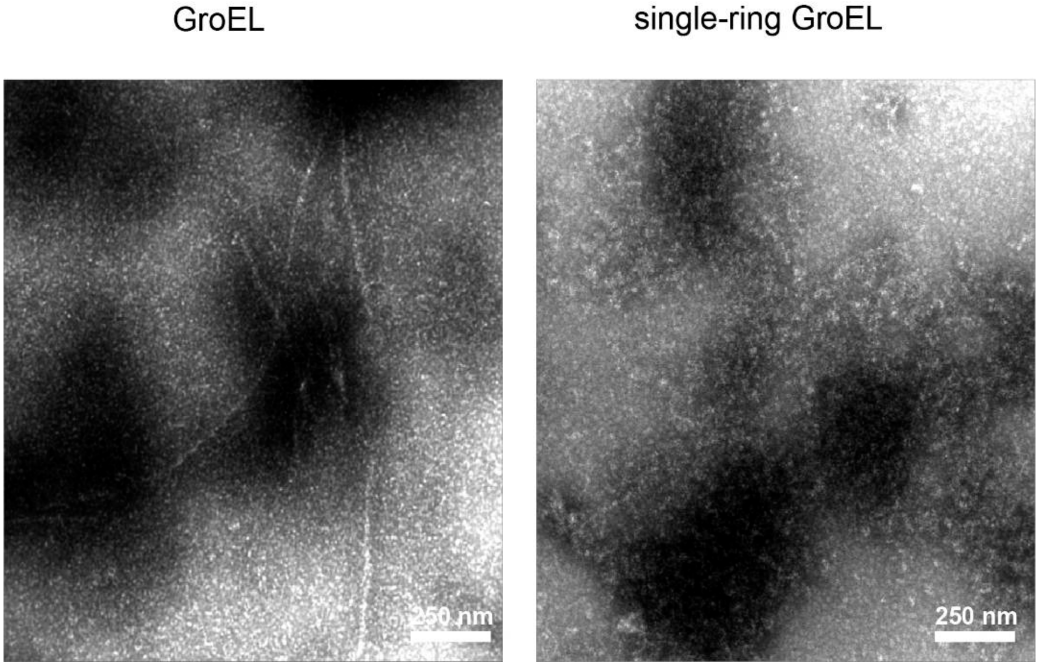

Supplement: Supplementary Information — Dataset 1 [file srep05614-s1.pdf]
